# Unravelling hidden battles: Genetic insights into parasite diversity and competition in mottled triplefin (*Forsterygion capito*)

**DOI:** 10.1007/s00436-025-08495-z

**Published:** 2025-06-10

**Authors:** Sila Viriyautsahakul, Robert Poulin, Sheri L. Johnson, Jerusha Bennett

**Affiliations:** Department of Zoology, University of Otago, PO Box 56, Dunedin, New Zealand

**Keywords:** *Forsterygion capito*, *Cardiocephaloides ovicorpus*, Trophic transmission, Helminth, Intraspecific competition, Parasite larval size

## Abstract

Parasites play an important role in ecosystems, often exhibiting complex life cycles that involve two to four host species and transmission through trophic interactions. By tracking parasite life cycles, predator–prey links can be identified, offering valuable insights into food webs. In this study, we examined the helminth community of the mottled triplefin (*Forsterygion capito*), one of the most common intertidal fish species in New Zealand, using molecular techniques. We identified 14 parasite species, including one new to science and three new host records. These findings established new predator–prey links in the previously known local food web. We also provide evidence of parasite competition, focusing on the trematode *Cardiocephaloides ovicorpus*, the most prevalent and abundant parasite found in the brain case of *F. capito*. As the intensity of *C. ovicorpus* metacercariae increased, their individual size decreased, suggesting intraspecific competition for resources within the limited space of the brain case. Additionally, *C. ovicorpus* may indirectly outcompete other parasite species using *F. capito* by inducing host behavioural manipulation to enhance its transmission to avian hosts given its encystment site within the brain case of the fish. Overall, our findings emphasise the importance of obtaining information about parasites in order to improve our understanding of species interactions within ecosystems.

**Supplementary Information:**

The online version contains supplementary material available at 10.1007/s00436-025-08495-z.

## Introduction

Studying parasites can provide valuable insights into ecosystem food web dynamics. Many aquatic parasites have complex life cycles, involving up to four host species within a single generation, with transmission often occurring through predator–prey interactions (Dunne et al. 2013; Fredensborg and Longoria 2012; Thompson et al. 2005). By tracing the transmission routes of parasites, predator–prey links in the food web can be identified, even without direct observation of predation—an especially challenging task in aquatic environments (Thompson et al. 2005; Bennett et al. 2023). Understanding parasite transmission routes can also reveal shifts in predator diets caused by climate change and human activities as well as the population dynamics of each host species (Marcogliese 2002; Byers 2021). Furthermore, tracking the life cycle of potential disease-causing parasites provides valuable information aiding the protection of species by improving the management and conservation of endangered host species involved (Randell and Bray, 1983; Jimenez-Uzcategui et al., 2015). However, marine ecologists often overlook parasites due to their small size, leading to a limited understanding of their life cycles, despite their high diversity, abundance, and essential role in shaping population dynamics and food web structure (Timi and Poulin 2020).

In New Zealand, knowledge of the parasite fauna remains largely incomplete, with approximately 96% of marine free-living taxa lacking records of their parasite assemblages (Bennett et al. 2022a). This gap extends to fish, where a large proportion of parasite species utilising fish as hosts have yet to be discovered and described, despite fish being important marine vertebrates that serve as intermediate and/or definitive hosts for various parasitic helminths (Bennett et al. 2022a; Lagrue et al. 2011). New Zealand is home to over 1387 species of marine fish, 19% of which are endemic, including some taxa entirely endemic to the country (Gordon et al. 2010). Triplefins (Family Tripterygiidae; sometimes referred to as cockabullies or kokopara) are amongst the most commonly encountered fish in New Zealand, with 10 genera and 26 species being endemic (Feary et al. 2009; Roberts et al. 2015). These small, benthic blennioid teleosts inhabit intertidal zones throughout the two main islands, as well as Stewart, the Chatham and Subantarctic Islands of New Zealand (Roberts et al. 2015).

In Otago Harbour, southern New Zealand, triplefins from the endemic genus *Forsterygion*, including the mottled triplefin (*Forsterygion capito*) and the common triplefin (*Forsterygion lapillum*), host the highest number of helminth species compared to other local taxa, according to a recent large-scale survey of parasite diversity and resolution of their life cycles across vertebrate and invertebrate taxa in the locality (Bennett et al. 2023). This includes 16 species from four taxa: cestodes, trematodes, nematodes, and monogeneans (Bennett et al. 2023). Most parasites were found in their larval stage, highlighting the importance of *Forsterygion* species as intermediate hosts of multiple parasite taxa. Moreover, several missing links in the local Otago Harbour food web as well as the large proportion of potentially undiscovered parasite species suggest that more parasites may use *Forsterygion* spp. as intermediate hosts in this locality (Bennett et al. 2022a, b, 2023).

Utilisation of the same intermediate host by multiple species of trophically transmitted parasites is a commonly observed scenario (Marcogliese 2002). However, the parasites often do not share the same definitive host, leading to conflict of interest and competition (Lagrue and Poulin 2008). Parasites may employ phenotypic manipulation of their intermediate host to increase the likelihood of successful transmission or even to reduce the transmission success of their competitors (Cézilly et al. 2000; Thomas et al. 2002). Alternatively, they may evolve to associate with the parasites sharing the same definitive host or avoid sharing intermediate hosts with other parasites with which they have a conflict of interest to reduce competition (Thomas et al. 1998, 2002).

Nevertheless, competition for resources within the intermediate host—both inter- and intraspecific—is often unavoidable, whether or not parasites share the same definitive host (Gower and Webster 2005; Mouritsen and Anderson 2017; Lagrue and Poulin 2008). This form of competition may be manifested by reductions in the body size of parasites. Generally, larger body size translates into higher individual fitness, enhancing survival and fecundity upon reaching maturity in the definitive host. Therefore, if space or other resources are limited within the intermediate host, a higher number of parasites in one host might result in smaller average individual size (Fredensborg and Poulin 2005; Saldanha et al. 2009; Dianne et al. 2012). However, only a few studies have previously investigated parasite competition, especially at intraspecific level, likely due to difficulties in finding a suitable case study. In mottled triplefins (*F. capito*), one of the 16 helminths discovered utilising the fish as a host is the trematode *Cardiocephaloides ovicorpus* (Dubois and Angel 1972). This trematode can reach high numbers in the same fish individual, and it is the only species recorded within the brain case of *Forsterygion* spp. in Otago Harbour, providing an opportunity to investigate intraspecific competition for limited space and resources within the brain case of the fish.

Therefore, in this study, we aimed to (1) genetically identify the helminths found in mottled triplefin (*F. capito*) to further explore the diversity of helminths and their transmission routes in Otago Harbour and (2) investigate intraspecific competition amongst *C. ovicorpus* within the limited space of the brain case of *F. capito*. We predicted a negative correlation between the intensity of infection and average size of *C. ovicorpus* per fish, as a larger number of parasites likely results in a higher degree of intraspecific competition.

## Method

### Sampling

The sampling took place on 13 February 2024 at Broad Bay (45°50′49.9″ S 170°37′23.5″ E), Portobello, Otago, New Zealand, where 34 mottled triplefins (*F. capito*) were captured by hand net during low tide. The fish were held in containers equipped with battery-operated aerators and transported back to the Department of Zoology, University of Otago.

### Dissection and parasite collection

The 34 fish were kept alive in aquaria for over 1 month and then euthanized using an overdose of Aqui-S (175 mg/L) following Animal Use Protocol (AUP) number 23–84, approved by the University of Otago Animal Ethics Committee. They were then freshly dissected. Each fish was measured (body length) to the nearest millimetre. For each fish, parasites were retrieved from within the brain case, eyes, gills, body cavity, and gastrointestinal tract, based on the likely infection sites recorded previously in *Forsterygion* spp. by Bennett et al. (2023). During the process of retrieving the brain, the epaxial muscle was cut through and therefore also examined. For the first individual of each new parasite morphotype found, a small piece of tissue was cut and stored in 100% ethanol for DNA sequencing. The rest of the body was stored in 70% ethanol as a voucher.

For most morphotypes, the parasites were present at low intensity (< 5 individuals per fish) and/or low prevalence (infecting ≤ 5 fish, maximum 15%). However, when parasites with the same morphology were present in more than five individuals per fish, two extra samples were preserved individually in 100% ethanol to be genetically tested to determine conspecificity of individuals. Additionally, when the prevalence of a morphotype was higher than five fish, three parasite individuals, one from each fish, were preserved in 100% ethanol to be genetically tested. All other parasite individuals were preserved in 70% ethanol.

### Parasite identification

Molecular techniques were utilised to sequence the parasite tissues and compare them with those in the GenBank® database. DNA was extracted using the DNeasy® Blood & Tissue Kit (Qiagen, Hilden, Germany), then amplified by polymerase chain reaction (PCR). For cestodes, trematodes, and monogeneans, the 28S rDNA gene was targeted using primers T16 and T30 (Harper and Saunders 2001), under conditions of 94 °C for 5 min, followed by 38 cycles of 94 °C for 30 s, 45 °C for 30 s, 72 °C for 2 min, and 72 °C for 7 min. For nematodes, the targeted region was the partial small subunit 18S rDNA gene using primers nem18SF and nem18SR using conditions from Wood et al. (2013). The PCR product was run in gel electrophoresis to visualise the amplified DNA. Samples with clear bands were cleaned using EXOSAP-TM™ Express PCR Product Cleanup Reagent (USB Corporation, Cleveland, OH, USA) and sent for sequencing by capillary electrophoresis at the Genetic Analysis Service of the Department of Anatomy, University of Otago, New Zealand.

The retrieved sequences were trimmed (default parameters) and manually edited to minimise incorrect and ambiguous base cells in Geneious Prime® v.2024.0.5 (https://www.geneious.com). The sequences were analysed in BLASTn searches on GenBank® to accurately identify the parasites to the lowest taxonomic level possible. For those with high prevalence or intensity, the sequences within each morphotype were aligned to examine whether they were conspecifics. However, we were not able to retrieve genetic sequences from one trematode morphotype. The species was instead identified to genus level based on morphology by an experienced taxonomist, Dr. Bronwen Presswell (Zoology Dept., University of Otago).

### Matching up trophic transmission routes

Some of our parasite sequences matched 100% with other sequences in GenBank, previously obtained from adult parasites within definitive hosts, during BLASTn searches. From this, we inferred that these matches indicate a trophic transmission route of the parasites from their intermediate host, *F. capito*, to definitive hosts via predation. We then categorised parasites into four definitive host groups—birds, elasmobranchs, teleosts, and mammals—to visualise the common host groups for these parasites as well as the abundance of parasites in each group.

### *Cardiocephaloides ovicorpus* size measurements and statistical analysis

To test whether intraspecific competition amongst *Cardiocephaloides ovicorpus* metacercariae within the brain case of triplefins results in smaller sizes, the parasites retrieved from the brain case (but not eyes) of each fish were photographed under a microscope under standard magnification. The metacercariae were released from cysts and relaxed in 45 °C ethanol to straighten the body muscles prior to measurement. The images were then analysed using ImageJ® v.1.54 g (Schneider et al. 2012) to measure the size of each *C. ovicorpus* metacercaria, taken as the length from the mouth (oral sucker) to the tail end. The largest and smallest sizes of the parasites in each fish were recorded, and the average size of *C. ovicorpus* in each fish was calculated; all measurements are in micrometres.

The sizes of *C. ovicorpus* (average, largest, and smallest) were statistically tested for their correlation with the total number of *C. ovicorpus* present in the brain per fish. For each of the three *C. ovicorpus* size measures, a linear model (LM) was performed in R version 4.4.0 (R core team 2023) using the packages *lme4* (Bates et al. 2015) and *lmerTest* (Kuznetsova et al. 2017), with the total number of the brain *C. ovicorpus* and fish size (body length) as the predictors. The data distributions met the assumption of normality for all responses (average, maximum, and minimum size); therefore, data transformation was not required.

## Results

### Parasite diversity

In total, we recovered 1406 helminth individuals from 34 *Forsterygion capito*. The parasites were collected from six different locations in the fish, including within the brain case, eyes, body cavity, gastrointestinal tract, gills, and flesh (epaxial muscle) (Table 1). We identified 14 distinct helminth morphospecies, comprising two nematodes, six cestodes (Fig. S1), five trematodes (Fig. S2), and one monogenean (Fig. S3). A full list of species with genetic sequences is provided in Table 2. Of these, 10 species had previously been reported in at least one species of triplefin (*Forsterygion* spp.) (Table 1). We also found a new host record for three parasite species including the nematode *Anisakis* sp. (Bennett et al. 2022b), cestode *Lacistorhynchus dollfusi*, and the trematode *Opecoelus* sp. 1 (Bennett et al. 2023). Notably, one of the trematodes, *Brevicreadium* sp., had their 28S sequences showing less than 81% similarity to any known sequences in the GenBank database.

**Table 1:** Location, life stage, maximum intensity, and prevalence of helminths found in mottled triplefin (*Forsterygion capito*) from Broad Bay, Portobello, New Zealand

Parasite species	Location	Life stage	Previously found in *Forsterygion* spp.	Total	Maximum intensity	%Prevalence
Nematoda						
*Hysterothylacium deardorffoverstreetorum*	Gastrointestinal tract	Larval	Yes	4	2	8.9
*Anisakis* sp. (Bennett et al. 2022b)	Body cavity	Larval	No	1	1	3
Cestoda						
Rhinebothriidae gen. sp. 1 & 2* (Bennett et al. 2023)	Gastrointestinal tract	Metacestode, plerocercoid type	Yes	25	11	20.6
Rhinebothriidae gen. sp. 3 (Bennett et al. 2023)	Gastrointestinal tract	Metacestode, plerocercoid type	Yes	107	57	26.5
*Acanthobothrium wedli*	Gastrointestinal tract	Metacestode, plerocercoid type	Yes	1	1	3
*Yamaguticestus* sp. 1 (Bennett et al. 2023)	Gastrointestinal tract	Metacestode, plerocercoid type	Yes	1	1	3
*Lacistorhynchus dollfusi*	flesh (epaxial muscle)	Metacestode, plerocercoid type	No	1	1	3.0
Cestoda spp.**	Gastrointestinal tract	Metacestode, plerocercoid type	NA	15	8	5.9
Trematoda						
*Opecoelus* sp. 1 (Bennett et al. 2023)	Gastrointestinal tract	Adult	No	1	1	3.0
*Macvicaria* sp.	Gastrointestinal tract	Adult	Yes	17	5	26.5
*Brevicreadium* sp.	Gastrointestinal tract	Immature adult	No	8	2	14.7
*Stephanostomum* sp.	Body cavity	Metacercariae	Yes	2	1	5.9
*Cardiocephaloides ovicorpus*	Brain case and eyes	Metacercariae	Yes	1224	233	100
Monogenean						
*Microcotyle* sp.	Gills	Adult	Yes	3	2	5.9

*Specimens were likely mixed during collection due to similar morphology; therefore, we combined the parameters of two species together here as specific numbers of either species could not be determined

**Two successfully sequenced specimens appeared to be two different species: Rhinebothriidae gen. sp. 2 (Bennett et al. 2023) and *Yamaguticestus* sp. 1 (Bennett et al. 2023). All 15 had identical morphology; therefore, they are grouped together and exclude specimens identified to species based on sequence data

**Table 2:** List of new 18S or 28S sequences (GenBank accession number) of helminths found in mottled triplefin (*Forsterygion capito*) from Broad Bay, Portobello, New Zealand

Parasite species	Isolate	18S	28S
Nematoda			
*Hysterothylacium deardorffoverstreetorum*	Fish1nem1	PV061862	-
*Anisakis *sp. (Bennett et al. 2022b)	Fish33nem2B	PV061863	-
Cestoda			
Rhinebothriidae gen. sp. 1 (Bennett et al. 2023)	Fish34ces1	-	PV521840
Rhinebothriidae gen. sp. 2 (Bennett et al. 2023)	Fish11ces2	-	PV521832
Rhinebothriidae gen. sp. 3 (Bennett et al. 2023)	Fish23ces3B	-	PV521836
*Acanthobothrium wedli*	Fish3ces4	-	PV521830
*Yamaguticestus* sp. 1 (Bennett et al. 2023)	Fish26ces5	-	PV521837
*Lacistorhynchus dollfusi*	Fish26 Tbrain1	-	PV521838
Trematoda			
*Opecoelus* sp. 1 (Bennett et al. 2023)	Fish11 tre1	-	PV521833
*Macvicaria* sp.	Fish20 tre2	-	PV521834
*Brevicreadium* sp.	Fish26 tre4	-	PV521839
*Cardiocephaloides ovicorpus*	Fish21eyeT	-	PV521835
Monogenean			
*Microcotyle* sp.	Fish6 tre3	-	PV521831

### Morphological variation

Some parasites exhibited distinct morphologies despite having identical genetic sequences. This included four distinct morphotypes of the intestinal cestode Rhinebothriidae gen. sp. 2 (Bennett et al. 2023) (Fig. S4). Interestingly, one of the morphotypes (Fig. S4 d) was shared between Rhinebothriidae gen. sp. 2 (Bennett et al. 2023) and *Yamaguticestus* sp. 1 (Bennett et al. 2023), as two successful sequences of morphologically identical specimens apparently represented these two different species. Consequently, we classified all cestodes with that morphotype as Cestoda spp. (Table 1). Additionally, we discovered two different morphotypes of the trematode *Cardiocephaloides ovicorpus* (Dubois and Angel 1972), with individuals found in the eyes exhibiting distinct morphology and smaller size compared to those found in the brain (Fig. S5).

### Parasite definitive hosts

**Fig. 1:**
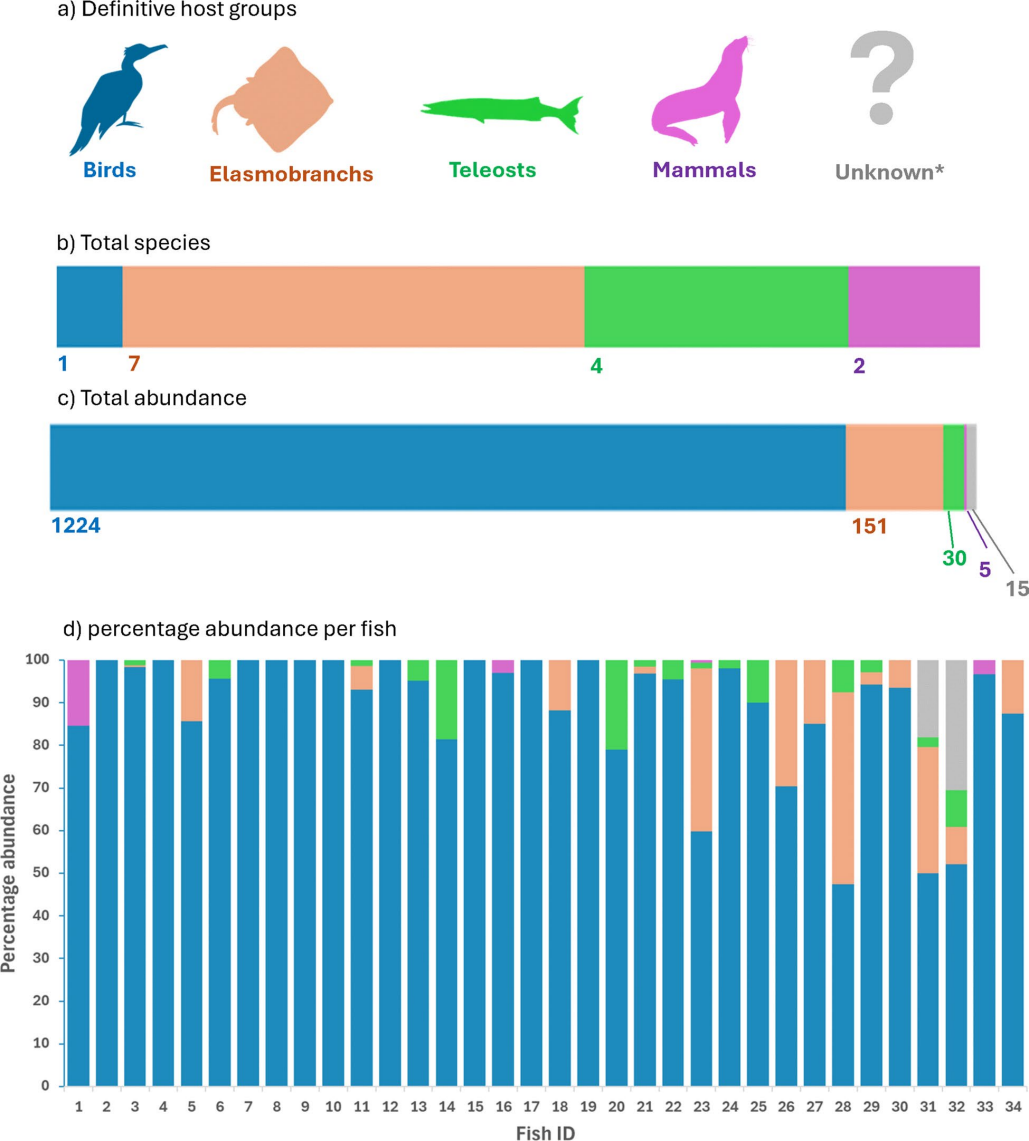
Total species and abundance of helminths collected from 34 mottle triplefins (*Forsterygion capito*) grouped by the parasites’ definitive host groups. *Including unidentified Cestoda spp., therefore including parasites that could not be assigned to host group

When grouping the helminths by their definitive hosts, we found that half of the parasite species use elasmobranchs as their definitive hosts, followed by teleosts as the second most common host (Fig. 1a, b). Most of these parasites were located free within the gastrointestinal tract of *F. capito*. However, their prevalence and intensity were generally low, with less than five infected fish for each species (< 15%) and/or fewer than five individuals per fish (Table 1). The trematode *Cardiocephaloides ovicorpus*, although it was the only species utilising birds as definitive host, had an exceptionally higher abundance than all other species or host groups, accounting for 87% of the total number of helminths found (Fig. 1c). Additionally, *C. ovicorpus* prevalence was 100% and made up no less than 50% of the total helminths retrieved from each fish, making it the most abundant helminth species found in this study (Fig. 1d).

### *Cardiocephaloides ovicorpus*

**Fig. 2:**
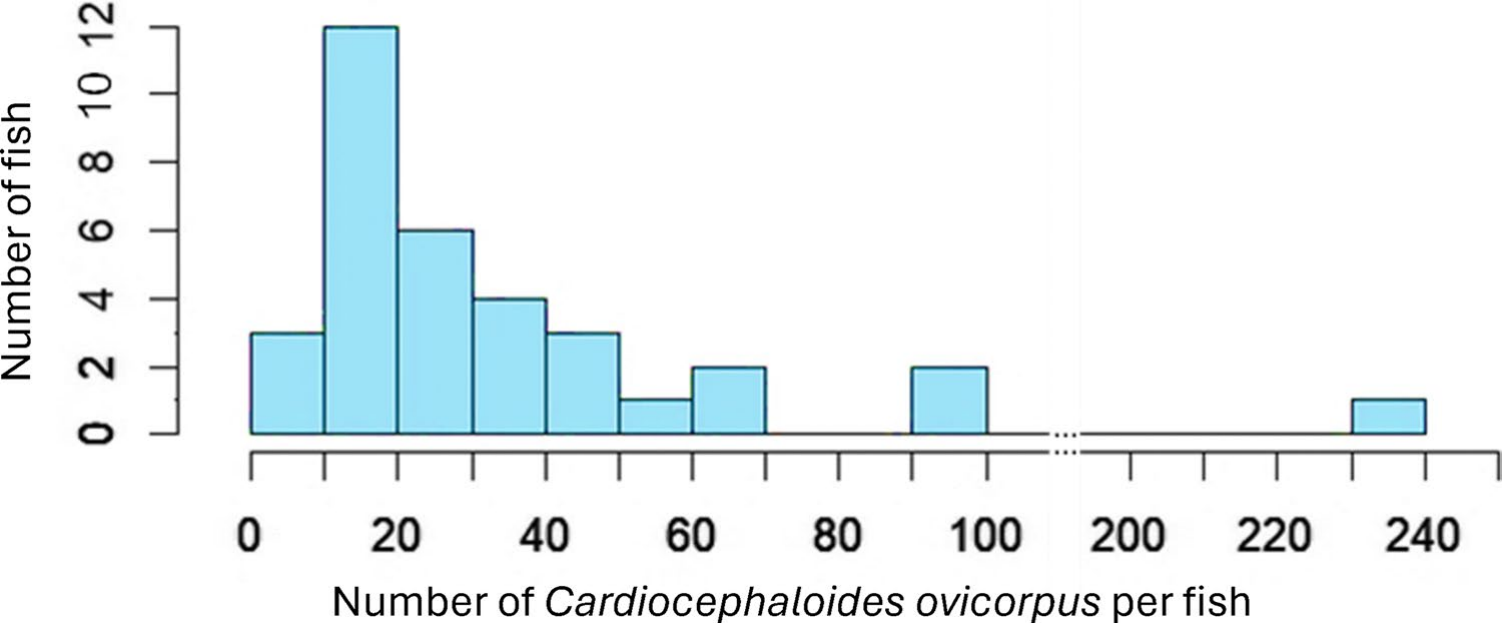
Frequency distribution of intensities of *Cardiocephaloides ovicorpus* infection amongst mottled triplefin (*Forsterygion capito*) (*N* = 34)

**Fig. 3:**
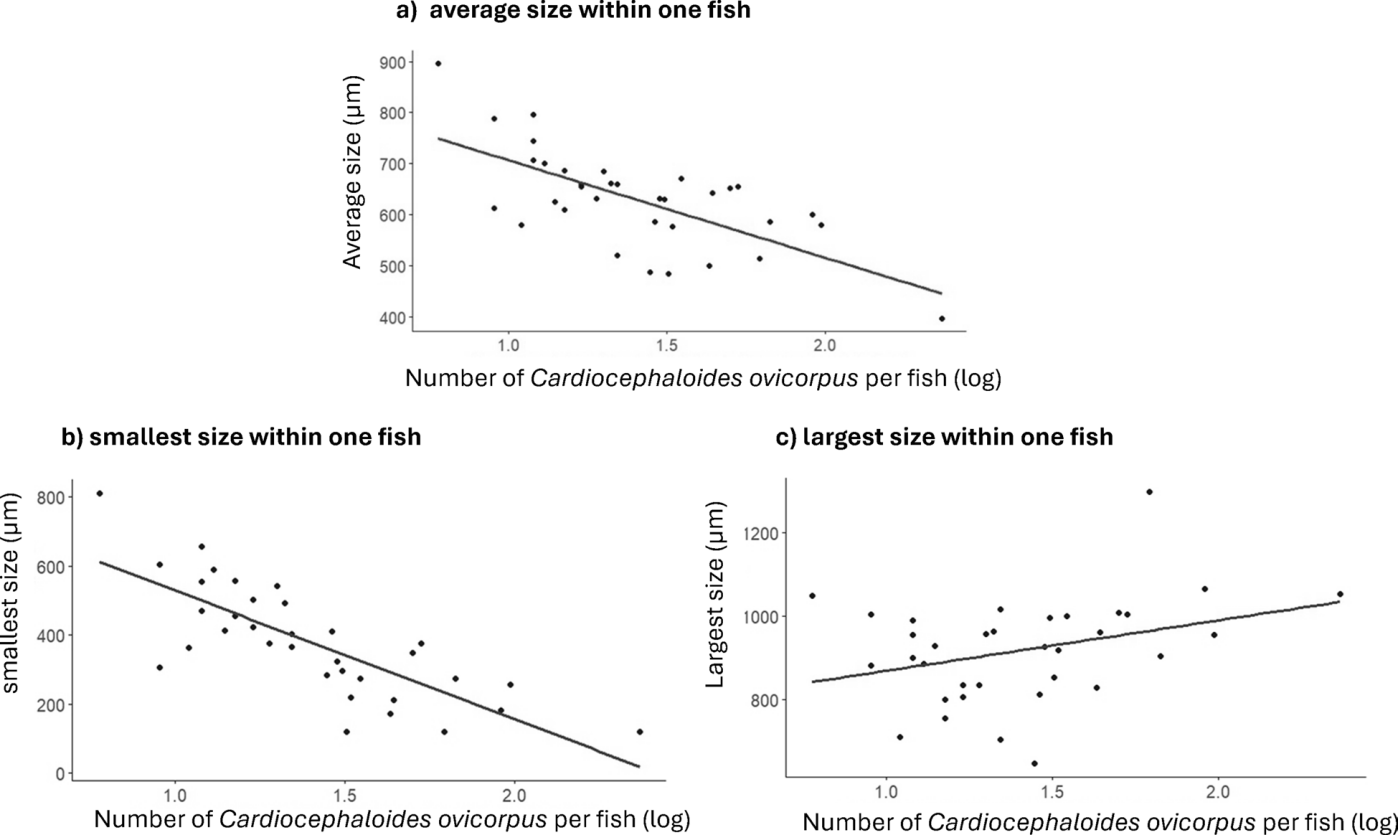
Correlation between total number of *Cardiocephaloides ovicorpus* within the brain case of mottled triplefin (*Forsterygion capito*) and **a** the average size of the parasites, **b** the size of the smallest individual, and **c** the size of the largest individual

A total of 1224 *C. ovicorpus* individuals were retrieved from 34 *F. capito* with the intensity ranging between 6 and 233 parasites per fish, and an average of 36 parasites per fish (Fig. 2). Of these, 1211 were recovered from the brain case, and the other 13 individuals were found within the eyes. Specifically, for those in the brain case, the average size (body length) of *C. ovicorpus* within a single host ranged from 397 to 897 µm. A linear model (LM) revealed a significant negative correlation between the average size of *C. ovicorpus* per fish and the total number of parasites (*t* = − 5.145, *P* < 0.001) (Fig. 3a). The size of the smallest *C. ovicorpus* within a single host also showed a significant negative correlation with the total number of parasites (*t* = − 4.319, *P* < 0.001) (Fig. 3b). In contrast, the correlation between the size of the largest *C. ovicorpus* within a single host and the total number of parasites was not statistically significant (*t* = − 1.006, *P* = 0.323) (Fig. 3c). Additionally, fish size was significantly positively correlated with both the average size (*t* = 2.507, *P* = 0.018) and the largest size (*t* = 2.753, *P* = 0.010) of *C. ovicorpus* within a host, though no significant correlation was found with the size of the smallest parasite within a fish.

## Discussion

We identified 14 genetically distinct helminth species from *F. capito*. While 10 had previously been recorded in *Forsterygion* spp. from Otago Harbour by Bennett et al. (2022b and 2023), we discovered three species as new host records for *F. capito* and one trematode likely represents a new species. We highlight the importance of genetic sequencing in species identification, as we observed considerable morphological variation amongst conspecifics, particularly cestodes, where up to four distinct morphotypes appeared to belong to a single species. Intraspecific morphological variation amongst parasites at the same life stage has been reported across various helminth groups and life stages and, indeed, poses challenges in parasite identification (Brazenor et al. 2018; Lloyd and Poulin 2012; Presswell and Bennett 2020). Nonetheless, these complications can be effectively resolved through molecular techniques.

**Fig. 4:**
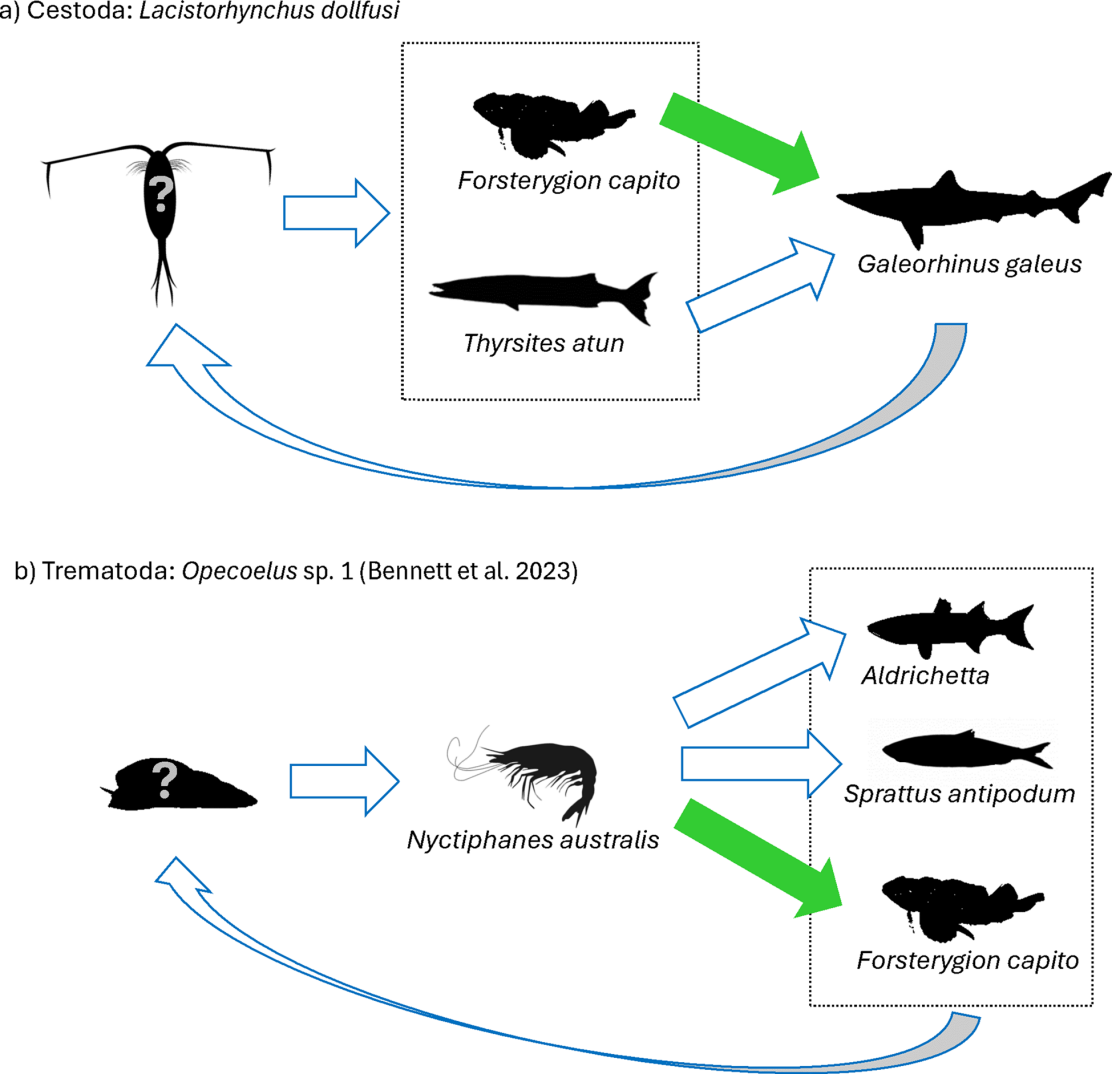
Newly established transmission routes (indicated by solid arrow) of two trophically transmitted helminths in Otago Harbour including **a** *Lacistorhynchus dollfusi* and **b** *Opecoelus* sp. 1 (Bennett et al. 2023). The first intermediate host(s) of both species still remain unknown

From our new host records of helminths, we were able to further resolve parasite transmission routes as well as to uncover two more predator–prey links in Otago Harbour. Firstly, this is the first evidence of the cestode *Lacistorhynchus dollfusi* (Fig. S1f) utilising *Forsterygion* spp. as an intermediate host (Fig. 4a). The complete life cycle of *L. dollfusi* has previously been elucidated in California, involving three hosts: a copepod, a teleost fish, and an elasmobranch (Sakanari and Moser 1989). In Otago Harbour, the species had been previously known to utilise barracouta (*Thyrsites atun*) as a second intermediate host and the school shark (*Galeorhinus galeus*) as a definitive host (Bennett et al. 2023). However, the first intermediate host, presumably a copepod, has yet to be found in this locality.

Secondly, this is the first discovery of *Opecoelus* sp. 1 (Bennett et al. 2023) (Fig. S2a) using *Forsterygion* spp. as another suitable definitive host (Fig. 4b). Based on other *Opecoelus* spp., their life cycles typically involve three hosts: a gastropod, a small crustacean, and a teleost (Cribb 1985). Previously, *Opecoelus* sp. 1 (Bennett et al. 2023) was found to utilise krill (*Nyctiphanes australis*) as a second intermediate host, and mullet (*Aldrichetta forsteri*) and New Zealand blueback sprat (*Sprattus antipodum*) as definitive hosts in Otago Harbour (Bennett et al. 2023). Our results suggest that this krill must make up a component of the diet of *F. capito* in some capacity for it to act as a definitive host. The first intermediate host of the species, however, has remained unknown. Overall, we have contributed to the development of the ecological food web of the Otago Harbour locality by using parasite transmission routes to confirm two more predator–prey links between the host species.

In contrast to the findings of Bennett et al. (2023), we recovered a much higher number of parasites per fish and a greater total abundance of the trematode *Cardiocephaloides ovicorpus*. Previously, the prevalence of *C. ovicorpus* was reported at approximately 40% of the fish sampled (only considering those collected from the same sampling site, Broad Bay), with numbers ranging from one to nine individuals per fish, which is significantly lower than what we observed in this study. Parasite abundance can be influenced by various factors. Host density and population size have been found to influence parasite population dynamics, which can fluctuate over time resulting in different abundance of parasites between two given sampling times (Arneberg et al. 1998; Bagge et al. 2004). In our case, a more likely scenario may be that the water temperatures in the months prior to our fish collection may have been generally warmer than in the period prior to the fish collection made by Bennett et al. (2023). Ambient temperature is known to promote trematode multiplication within the snail first intermediate host and the release of cercarial infective stages (Poulin 2006; Studer et al. 2010), leading to greater exposure and parasite acquisition by the second intermediate host, in this case triplefins. This raises some concerns for triplefin populations in the future due to global warming. According to New Zealand’s National Institute of Water and Atmospheric Research (NIWA, 2023), the country’s annual average temperature is rising, with warmer winter temperatures in the Otago Harbour locality likely providing more favourable conditions for parasite development and transmission.

Although the time interval between our study and the previous parasite survey by Bennett et al. (2023) was less than 5 years and in both cases sampling was conducted at approximately the same time of the year, this sharp increase in the number of *C. ovicorpus* in *F. capito* raises further concerns because one of the known definitive hosts of *C. ovicorpus* is the Otago shag (*Leucocarbo chalconotus*), a species endemic to New Zealand and highly localised to the Otago region (Presswell and Bennett 2021). Previously, high infection intensities of parasites from the genus *Cardiocephaloides* have been reported as a cause of mortality in their definitive bird hosts (Randell and Bray, 1983). Therefore, this increase may heighten concerns regarding management and conservation efforts to protect this rare endemic shag species from potential population declines.

Within the same host, parasites inevitably compete for limited resources to maximise fitness for reproduction upon reaching maturity, both inter- and intraspecifically (Fredensborg and Poulin 2005; Mouritsen and Anderson 2017; Lagrue and Poulin 2008). We do not consider that the few *C. ovicorpus* metacercariae found in the eyes of fish represent a case of ‘overflow’, with metacercariae settling in the eyes when conditions are too crowded in the brain. The reason is that eye metacercariae were mostly found in fish with relatively few brain metacercariae, whereas the fish most heavily infected by brain metacercariae had none in their eyes. For *C. ovicorpus* within the brain case of *F. capito*, we observed a significant negative correlation between the average size of the parasites per fish and the number of *C. ovicorpus* present, as predicted. Since *C. ovicorpus* was the only species encysting in the brain case of *F. capito*, this finding supports the idea that parasite size decreases as the consequence of a higher degree of intraspecific competition for limited space and resources within the brain case of the fish host. Therefore, we may conclude that intraspecific competition causes a decrease in fitness of the parasites. Additionally, the smallest parasites within individual fish also showed a negative correlation with the number of *C. ovicorpus* per host, further highlighting the action of competition and that its effects may be more serious for the smaller individuals.

Interestingly, the size of the largest parasites within a single fish was not correlated with the number of *C. ovicorpus* in the host brain, indicating that in all fish, some parasites were able to reach a certain size regardless of intraspecific competition level. The underlying reason for these patterns is not yet fully understood, it could be simply because those large parasites were the first individuals to infect the host and were thus able to achieve the largest size possible (Saldanha et al. 2009). Therefore, they did not suffer from the intense competition experienced by the later arrivals, a scenario supported by the trends observed in our study. Alternatively, the observed patterns may be due to stronger competition between individuals from different genotypes (i.e. parasites that were produced by different sporocysts, which developed in different first intermediate hosts), as parasites might compete less with genetically related individuals (Dianne et al. 2012; Parker et al. 2003). Future studies could explore this possibility by comparing genotypes between parasites of different sizes and those of similar sizes.

It is also worth noting that *C. ovicorpus* is the only species that utilises birds as definitive hosts amongst the 14 species of helminths found in this study (as illustrated in Fig. 1). Whilst the definitive hosts of other parasites is primarily elasmobranchs or predatory teleosts, *C. ovicorpus* likely faces not only intraspecific competition for resources but also interspecific conflicts of interests regarding the future of its triplefin host, as it has a different definitive host compared to the majority or any other parasites in *F. capito*. Many parasites are capable of manipulating the phenotype of their hosts to facilitate trophic transmission to a suitable definitive host. In that respect, *C. ovicorpus* has an advantage over other parasites of triplefins due to its high abundance, as shown in Fig. 1, where it dominated all other species within individual hosts. This suggests its specific impact on the host might eclipse that of other parasites. Additionally, the location of *C. ovicorpus* within the brain case of the fish may provide an additional advantage by placing the parasite in an ideal position to phenotypically manipulate the host, enhancing transmission success specifically from fish to bird and completing its life cycle (Fredensborg and Longoria 2012; Helland-Riise et al. 2020).

In conclusion, the mottled triplefin (*Forsterygion capito*) serves as an important second intermediate host for several helminths from various taxa, with at least 14 parasite species found in Otago Harbour (Southern New Zealand), with one representing a new species. We emphasise the importance of molecular techniques for parasite identification due to the morphological variability observed even amongst conspecific individuals. Additionally, we established two more aquatic predator–prey links in Otago Harbour by tracing parasite transmission routes, underscoring the value of parasites in enhancing our understanding of ecological systems. We also provide evidence of competition between parasites, using the brain-encysting trematode *Cardiocephaloides ovicorpus* as a model species. As parasite abundance increased, individuals displayed smaller average sizes, reflecting intraspecific competition for resources within the limited space of the fish braincase. Moreover, since *C. ovicorpus* is the only species in this system that transmits to birds, and given its privileged site of infection, we might expect it to have evolved host manipulation mechanisms, favouring its transmission to birds as opposed to unsuitable definitive hosts.

## Supplementary Information

Below is the link to the electronic supplementary material.

Supplementary file1 (PDF 294 KB)

## Data Availability

The dataset used for this study is available from https://figshare.com/articles/dataset/Triplefin_parasite_diversity_and_size_full_data_xlsx/28427864?file = 52399160.
