# Supplementary material for: Unravelling hidden battles: Genetic insights into parasite diversity and competition in mottled triplefin (Forsterygion capito)
Source: Parasitol Res. 2025 Jun 10;124(6):62. doi: 10.1007/s00436-025-08495-z (PMC12152018; doi:10.1007/s00436-025-08495-z)
Supplement: Supplementary file 1 — Supplementary file1 (PDF 294 KB) [file 436_2025_8495_MOESM1_ESM.pdf]

Unravelling Hidden Battles: Genetic Insights into Parasite Diversity and Competition in Mottled Triplefin (*Forsterygion capito*).

Sila Viriyautsahakul[1*], **Robert Poulin**[1], **Sheri L. Johnson**[1] and **Jerusha Bennett**[1]

[1] Department of Zoology, University of Otago, PO Box 56, Dunedin, New Zealand

*Corresponding author email: virlu009@student.otago.ac.nz, silaviriya@gmail.com

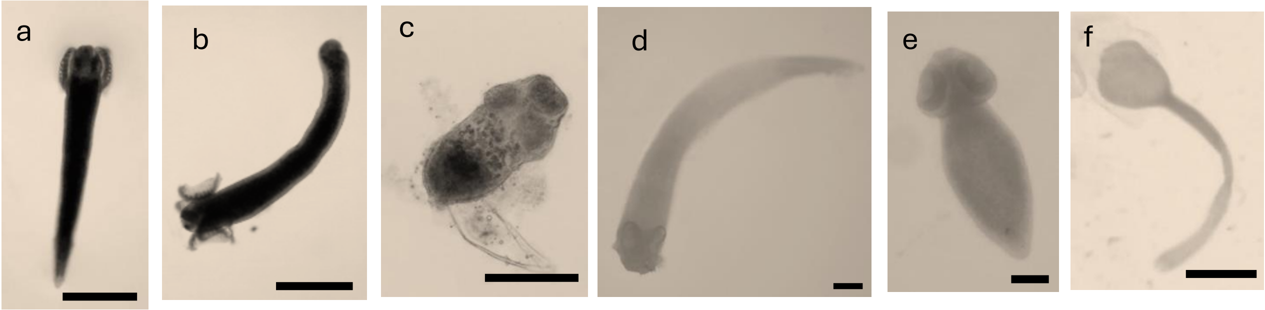

Figure S1. Cestodes found in mottled triplefin (*Forsterygion capito*) from Broad Bay, Portobello, New Zealand: (a) Rhinebothriidae gen. sp. 1 (Bennett et al. 2023). (b) Rhinebothriidae gen. sp. 2 (Bennett et al. 2023). (c) Rhinebothriidae gen. sp. 3 (Bennett et al. 2023). (d) *Acanthobothrium wedli.* (e) *Yamaguticestus* sp. 1 (Bennett et al., 2023). (e) *Lacistorhynchus dollfusi.* Scale bars: (a-b) 500 µm; (c-e) 200 µm; (f) 2 mm

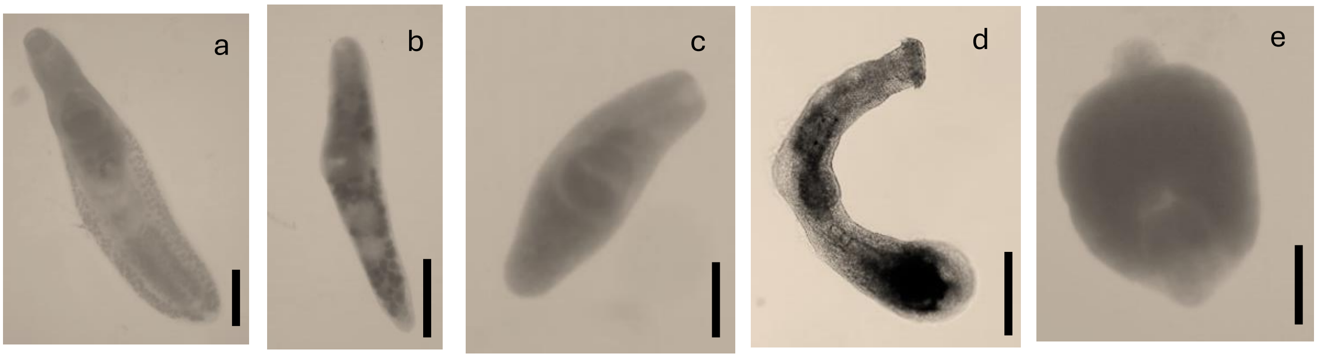

Figure S2. Trematodes found in mottled triplefin (*Forsterygion capito*) from Broad Bay, Portobello, New Zealand: (a) *Opecoelus* sp. 1 (Bennett et al. 2023). (b) *Macvicaria* sp. (c) *Brevicreadium* sp. (d) *Stephanostomum* sp. (e) *Cardiocephaloides ovicorpus*. Scale bars: (a-b) 500 µm; (c-e) 200 µm

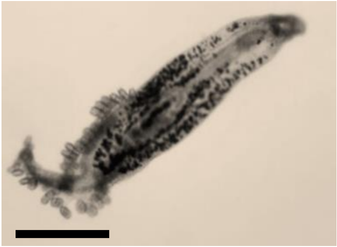

Figure S3. Monogenean *Microcotyle* sp. found in mottled triplefin (*Forsterygion capito*) from Broad Bay, Portobello, New Zealand. Scale bar is 500 µm

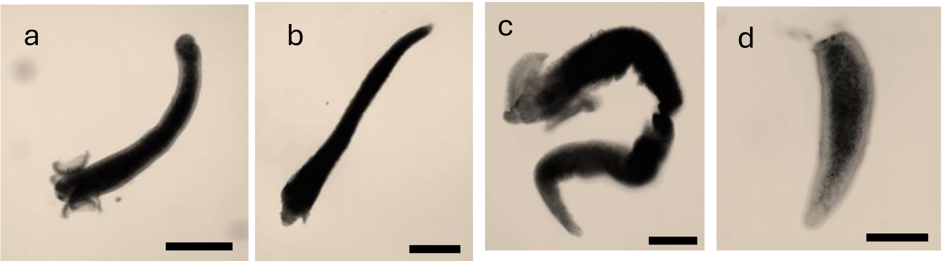

Figure S4. Four different morphologies of Rhinebothriidae gen. sp. 2 (Bennett et al. 2023) found in mottled triplefin (*Forsterygion capito*) from Broad Bay, Portobello, New Zealand. Scale bars: (a-c) 500 µm; (d) 200 µm

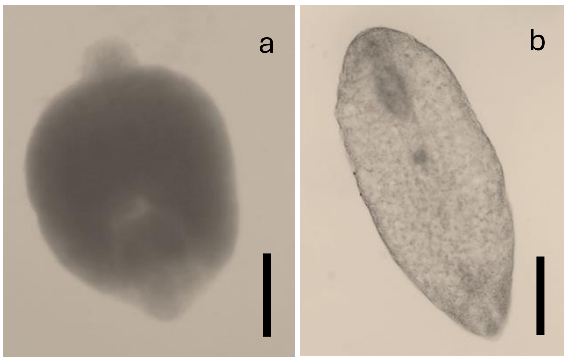

Figure S5. Two different morphologies of *Cardiocephaloides ovicorpus* found in (a) the brain, and (b) eyes of mottled triplefin (*Forsterygion capito*) from Broad Bay, Portobello, New Zealand. Scale bar is 200 µm
